# Why SWI? The sensitivity of susceptibility weighted imaging in aneurysmal subarachnoid haemorrhage in the chronic phase

**DOI:** 10.4102/sajr.v27i1.2520

**Published:** 2023-03-31

**Authors:** Yeshkhir Naidoo, Rohen Harrichandparsad, Khatija Amod

**Affiliations:** 1 Department of Radiology, Faculty of Diagnostic Radiology, University of KwaZulu-Natal, Durban, South Africa; 2 Department of Neurosurgery, Faculty of Neurosurgery, University of KwaZulu-Natal, Durban, South Africa; 3 Department of Radiology, Inkosi Albert Luthuli Central Hospital, University of KwaZulu-Natal, Durban, South Africa

**Keywords:** aneurysmal subarachnoid haemorrhage (ASAH), susceptibility weighted imaging (SWI), chronic aneurysmal subarachnoid haemorrhage, endovascular coiling, haemosiderosis, intracranial aneurysm

## Abstract

**Background:**

Incidentally detected unruptured intracranial aneurysms have a prevalence of 3% with some predisposed to rupture and others remaining static. Diagnostic knowledge of previous aneurysmal subarachnoid haemorrhage (ASAH) in the chronic phase could identify patients requiring treatment.

**Objectives:**

To assess the sensitivity of susceptibility weighted imaging (SWI) in the detection of ASAH at 3 months post ictus and determine any influencing effects.

**Method:**

A retrospective chart analysis of 46 patients with ASAH who underwent post-embolisation SWI imaging at 3 months. The SWI and available initial CT brain scans or CT reports were evaluated and correlated with patient demographics and clinical severity.

**Results:**

Susceptibility weighted imaging indicated a sensitivity of 95.7% in the detection of ASAH at 3 months. Increased number of haemosiderin zones on SWI correlated with older patient age (*p* = 0.0003). Clinical severity (World Federation Neurosurgical Societies Score) showed a tendency towards a statistically relevant relationship (*p* = 0.07). No statistically significant relationship was identified between the number of haemosiderin zones and initial CT modified Fisher score (*p* = 0.34) or the causative aneurysm location (*p* = 0.37).

**Conclusion:**

Susceptibility weighted imaging is sensitive in the detection of ASAH at 3 months, increasing in sensitivity with patient age and higher initial clinical severity.

**Contribution:**

In patients presenting in the subacute to chronic phase with a clinically suspicious history of previous aneurysm rupture but without convincing CT or spectrophotometry evidence, SWI can detect previous rupture. This can identify patients who could benefit from endovascular treatment and those who can safely undergo follow-up imaging.

## Introduction

Patients presenting in the subacute to chronic phase with a clinically suspicious history of previous aneurysm rupture, but without convincing radiological or spectrophotometry evidence, present a clinical conundrum to patient management. The natural history of intracranial aneurysms varies with some inherently being predisposed to rupture (5 year risk of 3.4%), while most others remain unruptured and without complications.^1,2^ The treatment of ruptured aneurysms is well established because of the increased risk of re-rupture and associated high patient mortality and morbidity.^3^ Preventative repair is not without risks (risk of 6% – 10% of poor neurological outcome) and should be weighed against any potential benefit.^4^ It is therefore important to differentiate a previously ruptured from an unruptured intracranial aneurysm.

In the acute phase, non-enhanced CT is the modality of choice for aneurysmal subarachnoid haemorrhage (ASAH) with sensitivities initially at 98%, reducing to 50% at 1 week and 0% after 3 weeks.^5^ The decreasing sensitivity over time is attributable to CSF flow dynamics clearing the initial haemorrhagic load and thereby reducing its conspicuity on CT. However, CT plays no role in the detection of subacute and chronic ASAH and other investigations such as lumbar puncture CSF spectrophotometry and MRI are helpful. Lumbar puncture CSF spectrophotometry detects bilirubin, indicative of previous subarachnoid haemorrhage, which remains positive up to 14 days following ASAH with a subsequent rapid decrease in sensitivity.^6^ These factors limit the usefulness of CSF analysis for the detection of ASAH in the subacute to chronic phase.

Pathophysiologically, after the onset of subarachnoid haemorrhage, a clot forms at the rupture site while the remaining free erythrocytes are either absorbed by the arachnoid villi and re-enter the vascular system or undergo lysis, resulting in free haeme deposition on subpial surfaces and conversion to intracellular haemosiderin.^7,8,9^ The initial haematoma surrounding the rupture is eventually washed away or phagocytosed by macrophages and converted to haemosiderin, accounting for greater volume of haemosiderin staining at the site of aneurysmal rupture, which is resistant to the effects of CSF flow and can be detected well into the chronic period as demonstrated by autopsy studies.^10^

In the subacute phase of ASAH, fluid-attenuated inversion recovery (FLAIR), T2*, double inversion recovery and SWI are all useful. These sequences detect deoxyhaemoglobin which is paramagnetic and takes up to 90 days to clear. Any residual blood products are converted to haemosiderin.^10^ Studies have even shown that after 3 months, haemosiderin remains unchanged for up to 16 years on T2* imaging.^11,12^ Haemosiderin staining along the superficial central nervous system is defined as superficial siderosis and has traditionally been because of repeated chronic subarachnoid haemorrhages of any cause. Recent studies with pathology specimens confirm that superficial siderosis can occur in nearly all patients after a single episode of ASAH.^12^

Imaging options for ASAH in the chronic phase have been limited with a few studies demonstrating the utility of T2*, with sensitivities ranging between 54.2% and 89.9%.^13,14,15,16^ Apart from the wide variation in sensitivity, T2* is limited because of its inability to differentiate between haemosiderin and calcification.^10,12,13^ Susceptibility weighted imaging utilises inherent susceptibility variations such as T2* but has the improved benefit of high resolution and allows differentiation between haemosiderin and calcification.^10,12,13^ In addition, SWI has improved sensitivity of four times that of T2* in the detection of intraparenchymal haemorrhage because of its use of phase imaging.^15^ Susceptibility weighted imaging has not been compared with T2* in the assessment of extra-axial haemorrhage and it can only be presumed to exceed T2* sensitivity.

To our knowledge, no published study has investigated SWI’s utility in detecting haemosiderin in the chronic phase of ASAH (after a single haemorrhagic event as assessed clinically). The aim of this study was to determine the ability of SWI to detect ASAH at 3 months post ictus, investigate factors influencing the amount of haemosiderin staining and ascertain if haemosiderin staining identified on SWI could correlate with the initial aneurysm location.

## Methods

### Study population

**FIGURE 1:**
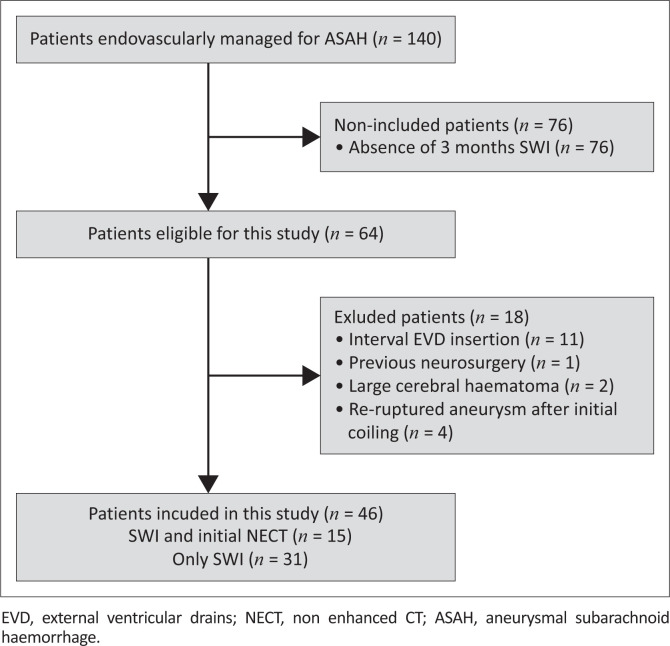
Flow chart of patient selection.

This was a retrospective, analytic, descriptive study of patients with ASAH managed at Inkosi Albert Luthuli Hospital, KwaZulu-Natal, between 01 June 2015 and 30 June 2019. The neurosurgical department offers services for both endovascular and microsurgical clipping of ASAH. Patients who underwent confirmatory initial CT brain imaging shortly after ASAH at their base hospitals and subsequent endovascular repair of the causative aneurysm were considered. Of these, all patients who underwent the same routine MRI brain sequences (including SWI) at 3 months post endovascular repair were included (Figure 1). Patients who had previous intracranial surgery, haemorrhage, trauma or malignancy prior to the initial ASAH or between the repair of the causative aneurysm and the acquisition of the SWI study were excluded.

### Data collection

Patient demographics and clinical information were obtained using the hospital information system. Patient charts were reviewed for date of ictus, initial CT report, modified Fisher score, age, gender, date of CT and SWI, World Federation Neurosurgical Societies Score (WFNS), date of endovascular surgery, location of aneurysms on digitally subtracted angiograms and any clinical information, which precluded involvement in the study.

The radiology information system and picture archive and communication system were used to analyse the available CT scans from base hospitals (*n* = 15) and all MRI SWI scans performed at our institution (*n* = 46) with a 3T Siemens machine, using the axial 5 mm and 3 mm slices, respectively. All confirmatory NECT were performed at the patients’ referral hospitals where the diagnoses were made. Where these CT scans were available, those images were evaluated in the study. The CT and SWI images were analysed by a senior radiologist with over 25 years of experience and an interest in neuroradiology as well as by a senior radiology registrar. The CT and MRI images were interpreted in separate settings individually and performed as blind reads. Available CT and MRI images were read by the two readers independently with a combined reading performed together for discussion and conformity.

The subarachnoid space was divided into 13 zones (adapted from prior studies that analysed acute phase ASAH with SWI^7,8^) – six were peripheral zones: frontoparietal, temporooccipital and Sylvian fissure (divided into a right and left side), five were central zones: inter-hemispheric and ventricular system (right and left lateral, 3rd and 4th ventricles) and the remaining two zones were in the perimesencephalic and posterior cranial fossa (defined as the prepontine, superior cerebellar and cisterna magna). Haemosiderin staining was defined as blooming with corresponding aliasing on phase images, whenever identified in the specified sulcal/ventricular surfaces. If a localised maximum site of haemosiderin staining was identified, with the greatest amplitude of blooming, its location was recorded. Modified Fisher scores were graded on the available initial NECT scans and where not available, were calculated from the radiology CT report findings and compared with the neurosurgical recorded modified Fisher scores.

### Statistical analysis

Statistical analyses were performed using the Statistical Package for Social Science (SPSS) version 26.0 to obtain the means, frequencies and perform a univariate analysis. Level of statistical relevance used was *p* < 0.05.

### Ethical considerations

Ethics approval received from Higher Research Committee at Inkosi Albert Luthuli Central Hospital (clearance number: BREC/00001655/2020).

## Results

The final study cohort included 46 patients with ASAH who were managed at Inkosi Albert Luthuli Hospital with endovascular aneurysmal repair and who underwent SWI imaging at 3 months post ictus.

### Haemosiderin Zones

SWI detected haemosiderin in 44/46 (95.7%) patients with ASAH. Of the two patients who had no haemosiderin staining on SWI at 3 months, the first patient had a right posterior communicating aneurysm, modified Fisher score 1, WFNS grade 2, and was 57 years old. The second patient had an anterior communicating aneurysm, modified Fisher score 1, WFNS grade 1 and was 28 years old. A total of 14 (30.4%) patients had multiple intracranial aneurysms.

Demographic data relating to haemosiderin zones are presented in Table 1. Females represented 63% (*n* = 29) of patients. The median age was 49 years (range 17–69 years). A statistically significant correlation was identified between increasing number of haemosiderin zones and increasing age (*p* = 0.003). No relationship was found between gender and the number of haemosiderin zones.

**TABLE 1:** Correlation of demographic, clinical and radiological factors affecting the extent of haemosiderin zones.

Factors affecting haemosiderin detection on SWI	Number of haemosiderin zones						*p*-value
	< 6 (*n* = 25)			7–13 (*n* = 22)			
	*n*	%	Mean ± s.d.	*n*	%	Mean ± s.d.
**Demographics**	-	-	-	-	-	-	-
Age	-	-	43.3 ± 12.3	53.8	-	53.8 ± 10.7	0.003*
**Age group**	-	-	-	-	-	-	0.05
17–40	11	44.0	-	3	13.6	-	-
41–54	9	36.0	-	9	40.9	-	-
55–69	5	20.0	-	10	45.5	-	-
**Gender**	-	-	-	-	-	-	0.9
Male	9	36.0	-	8	36.4	-	-
Female	16	64.0	-	14	63.6	-	-
**Clinical WFNS**	-	-	-	-	-	-	0.07**
1	19	79.2	-	12	54.5	-	-
2	3	12.5	-	3	13.6	-	-
3	0	0.0	-	4	18.2	-	-
4	2	8.3	-	3	13.6	-	-
**Radiological**	-	-	-	-	-	-	0.34*
Modified Fisher score (*n* = 39)	-	-	-	-	-	-	-
< 3	4	20.0	-	1	5.0	-	-
≥ 3	16	84.2	-	19	90.5	-	-
**Circulation aneurysm**		-	-	-	-	-	0.37***
Anterior	16	69.6	-	12	54.5	-	
Posterior	7	30.4	-	10	45.5	-	-

WFNS, World Federation Neurosurgical Societies.

* , Two-sample *t*-test with equal variances.

** , Two-sample Wilcoxon rank-sum (Mann–Whitney) test.

*** , Fisher’s exact test.

There was a tendency towards a higher number of haemosiderin zones having higher initial clinical severity, WFNS; however, not reaching statistical significance (*p* = 0.07). Five patients had a modified Fisher score < 3 and 34 patients had modified Fisher scores of 3–4. No statistical significance was found between the number of haemosiderin zones and the type of circulation aneurysm (*p* = 0.37) or the modified Fisher score (*p* = 0.34).

### CT versus MRI

In those patients with both SWI and available initial NECT images (15 patients), 390 zones were analysed (195 CT and 195 SWI zones). Of the total 195 zones on CT, 86 positive haemorrhage zones were identified. Of the total 195 zones on SWI, 108 positive haemorrhage zones were detected (Table 2). Overall, SWI (108 regions) detected more regions than CT (86 regions) with a global sensitivity of 61% and specificity 51% per location (Table 3).

**TABLE 2:** Correlation between areas of subarachnoid haemorrhage by modality.

Modality	Location									Total
	FPC	TOC	IHF	SVF	PMC	PFC	Intraventricular			
							Lateral ventricle	3rd ventricle	4th ventricle	
CT	16	3	10	18	12	7	13	2	5	86
SWI	18	10	10	21	7	11	21	1	9	108

FPC, frontoparietal cistern; TOC, temporo-occipital cistern; IHF, interhemispheric fissure; SVF, Sylvian fissure; PMC, perimesencephalic; PFC, posterior fossa cisterns; SWI, susceptibility weighted imaging.

**TABLE 3:** Location-based sensitivity and specificity of SWI.

Location	Sensitivity	Specificity	PPV
**Convexity FP**			
Right	0.63	0.43	0.56
Left	0.63	0.43	0.56
**Convexity TO**			
Right	0.00	0.64	0.00
Left	0.50	0.62	0.17
**Sylvian cistern**			
Right	0.71	0.38	0.50
Left	0.91	0.50	0.83
**lH**	0.70	0.40	0.70
**Perimesencephalic**	0.50	0.67	0.86
**PCF**	0.86	0.38	0.55
**LV**			
Right	1.00	0.50	0.64
Left	0.83	0.44	0.50
**3V**	0.00	0.92	0.00
**4V**	0.67	0.33	0.40

**Total**	**0.61**	**0.51**	-

FP, frontoparietal; TO, temporo-occipital; IH, inter-hemispheric; PCF, posterior cranial fossa; LV, lateral ventricle; PPV, positive predictive value.

### Localisation of aneurysm

Maximum haemosiderin staining localised to one zone was found in 73.9% (*n* = 34) while the remainder demonstrated either generalised haemosiderin staining (*n* = 10) or no haemosiderin staining (*n* = 2). Localisation for each of the zones were as follows: Sylvian cistern 47.1% (*n* = 16), interhemispheric fissure 41.2% (*n* = 14), perimesencephalic 5.9% (*n* = 2) and posterior cranial fossa cisterns 5.9% (*n* = 2).

Maximum haemosiderin staining within the Sylvian cistern was because of five aneurysms of the middle cerebral (100% of all middle cerebral artery (MCA) aneurysms; PPV = 31.25%), eight of the posterior communicating (61.53% of all posterior communicating artery aneurysms; PPV = 50%) and three of the distal internal carotid arteries (100% of all distal internal artery aneurysms; PPV = 18.75%).

Maximum haemosiderin staining in the interhemispheric fissure was isolated to 12 aneurysms of the anterior communicating (70.59% of all anterior communicating artery aneurysms; PPV = 85.71%) and two of the pericallosal arteries (100% of all pericallosal aneurysms; PPV = 14.29%).

Maximum haemosiderin staining in the perimesencepahlic cistern was limited to two aneurysms of the posterior communicating arteries (15.38% of all posterior communicating artery aneurysms; PPV = 100%). Maximum haemosiderin staining in the posterior cranial fossa cistern was isolated to one aneurysm of the posterior communicating (7.69% of all posterior communicating artery aneurysms; PPV = 50%) and one posterior inferior cerebellar artery aneurysm (100% of all posterior inferior cerebellar artery aneurysms; PPV = 50%).

## Discussion

**FIGURE 2:**
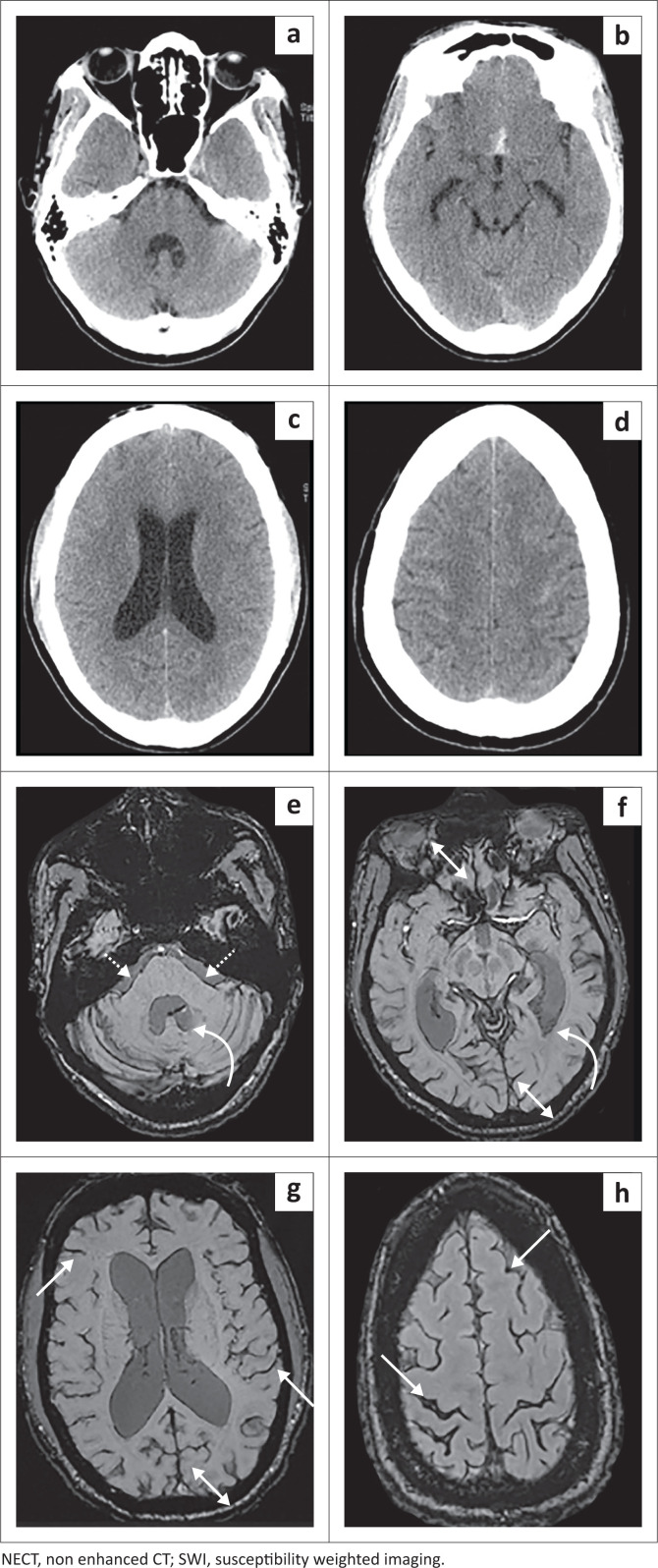
The NECT at presentation (a–d) and SWI at 3 months (e–h) in a patient with a ruptured anterior communicating aneurysm. The NECT demonstrates localised haematoma within the lamina terminalis (a) and frontoparietal convexities (d). The SWI at 3 months demonstrates diffuse blooming (haemosiderin) remote to the initial haemorrhagic load now along the cerebral convexities (white arrows), interhemispheric fissure (double white arrows), intraventricular (curved white arrow) and posterior cranial fossa cisterns (dashed white arrows) (e).

This study demonstrated the ability of SWI to detect ASAH in the chronic phase (> 3 months) after a single aneurysmal haemorrhage: 95.7% (44/46) as depicted in Figure 2. These findings exceeded previous studies utilising T2* in the same setting with rates of only 54.2% – 89.9%.^13,14,15,16^ This is likely attributable to SWI employing phase differences, which may not necessarily correspond to noticeable T2* effects, as shown in intraparenchymal haematomas demonstrating increased sensitivity of up to 4× in SWI.^15^

Of the two patients who were negative for haemosiderin staining on SWI, a low modified Fisher score of 1 and low WFNS of 1–2 were mutual findings. It is postulated that smaller initial haemorrhagic loads may be less likely to have haemosiderin staining detectable on SWI in the chronic phase. This corresponds with prior studies conducted by Lummel et al. and Falter et al. in patients with chronic ASAH and T2* who similarly showed absent haemosiderin staining in patients with lower modified Fisher scores.^12,14^

### Impact of location on SWI detection of aneurysmal subarachnoid haemorrhage

#### Convexity

Overall, SWI detected more regions of haemorrhage than the initial CT (as depicted in Figure 2) with a location-based sensitivity of 56% and specificity of 50%. A previous study by Mulé with T2* showed a similar sensitivity of 64% with an improved specificity of 82%.^16^ The marked difference in specificity was unanticipated and thought to be because of CSF flow dynamics causing haemorrhage to be washed to more regions remote from the site of rupture and hence detected on imaging in the chronic phase.^12,13,16^

#### Basal Cisterns

**FIGURE 3:**
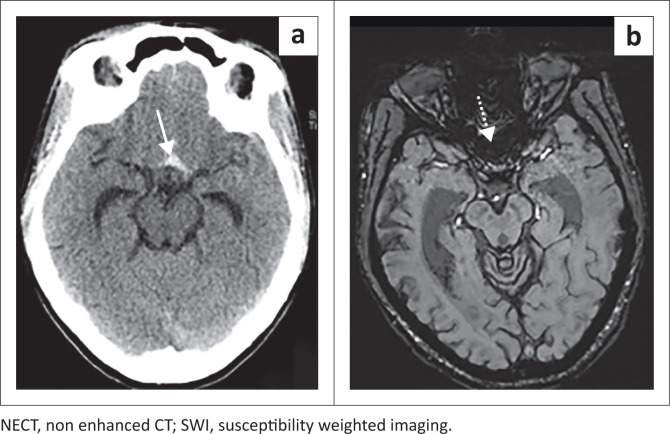
The NECT and SWI in patient with a ruptured anterior communicating aneurysm. Subarachnoid haematoma (a. white arrow) in the suprasellar cistern is not appreciated on the 3 month SWI because of adjacent susceptibly artefact from the base of skull (b. white dashed arrow).

This study showed a sensitivity of 50% in the perimesencephalic cistern, exceeding that of previous studies with T2* of 8%.^16^ The poor sensitivity in this region was because of regional artefact from the base of skull and high CSF flow displacing any haemorrhage as depicted in Figure 3.^12,13,16,17,18^

#### Ventricular System

**FIGURE 4:**
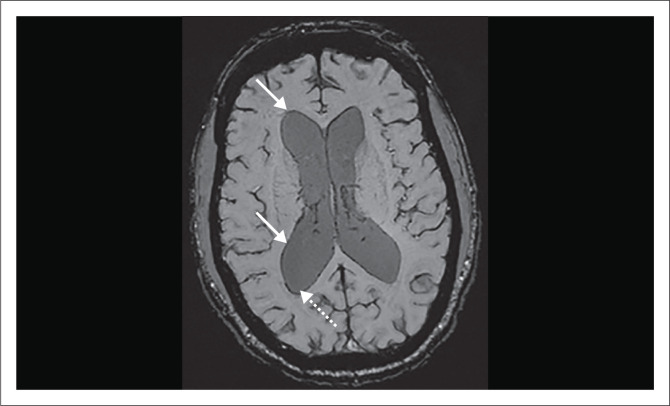
Susceptibility weighted imaging in a patient with a ruptured anterior communicating aneurysm. Haemosiderin staining the ependymal surface of the ventricles (white arrows), predominantly involving the dependent occipital horns (dashed white arrow).

Previous T2* studies in chronic ASAH showed poor sensitivities in the detection of haemorrhage in the ventricular system. Mulé et al. demonstrated a sensitivity of 10%.^16^ This was thought to be related to high CSF flow velocities in these locations, preventing adequate time for haemosiderosis.^12,13,16^ The current study contradicted this with increased sensitivity of 62.5% for haemosiderin, even exceeding regions seen on the initial NECT as depicted in Figure 4. These areas of haemosiderin identified on SWI were located in the dependent regions of the ventricular system (occipital horns of the lateral ventricles and dependent lateral recesses of the 4th ventricle).

Two postulated reasons were thought to account for this. Firstly, the inherent increased sensitivity and high-resolution imaging of SWI compared with T2* resulted in a greater sensitivity. Secondly, over time there is reflux of haemorrhage from the cisterns into the ventricular system in patients with subarachnoid haemorrhage as shown in previous studies.^8,19^ This process is thought to occur to equalise CSF pressures and may show haemosiderin in the ventricular system in patients who initially did not have intraventricular haemorrhage.

#### Infratentorial

Studies by Falter et al. and Mulé et al. showed T2* detection rates of haemosiderin in chronic ASAH in the infratentorial cisterns approaching 70% – 90%.^12,16^ Despite this, Lummel et al. and Mulé et al. demonstrated poor sensitivity of haemosiderin on T2* in the infratentorial compartment 10% – 21.9%.^14^ Susceptibility artefact in these sequences is related to the strength of the MRI magnetic field and previous surgical washout. However, Lummel et al. utilised a 3T machine similar to the other studies and the majority of patients underwent endovascular repair. A reason to account for the discrepancy in these studies is unknown.

**FIGURE 5:**
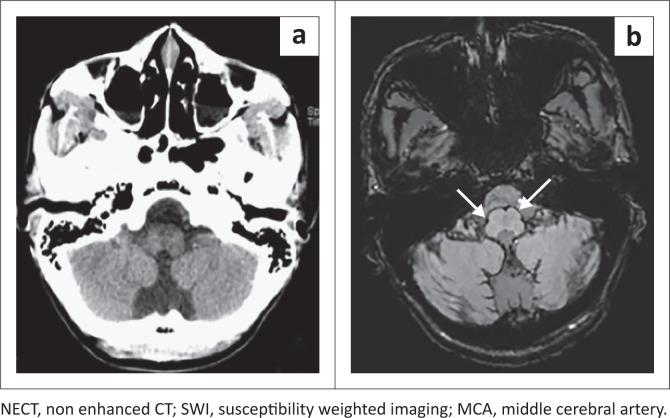
The NECT (a) and SWI (b) in a ruptured right MCA aneurysm. SWI at 3 months demonstrates haemosiderin staining (white arrows) on the pial surface, not seen on initial NECT.

In comparison, the current study showed high haemosiderin detection, with a sensitivity of 86% in the infratentorial compartment, exceeding that of the initially detected haemorrhage on NECT (SWI 11; CT 7) as depicted in Figure 5. This is most likely because of CSF flow as described by Koeppen et al., who showed in CSF flow studies that the brainstem and cerebellar convexities initially receive preferential CSF flow and serve as a site of haemosiderin deposition, albeit remote to the site of rupture. This has been described in patients with recurrent subarachnoid haemorrhage causing superficial haemosiderosis.

### Factors contributing to haemosiderin detection on SWI

Secondary objectives of the study were to evaluate for any correlations between the number of haemosiderin-stained zones with patient demographics, location of the causative aneurysm, initial clinical (WFNS) or radiological severity (modified Fisher score).

#### Demographics

Studies performed by Imaizumi et al. and Falter et al. showed no statistical significance between age and haemosiderin extent on T2* imaging. However, previous researchers have shown that age had a statistically significant relationship with the presence of haemosiderin on T2* GE imaging (*p* = 0.02).^12,13,14^ Like previous studies it is hypothesised that with ageing and subsequent cerebral atrophy there are larger subarachnoid spaces, which allow for larger areas of haemorrhage aggregation and hence haemosiderin deposition.^12^ The current study concurred with Lummel et al. indicating a statistically significant correlation with increasing numbers of haemosiderin deposition zones and increasing patient age (*p* = 0.003*).*

Aneurysmal subarachnoid haemorrhage has a 50% increased incidence in the female population. Previous studies utilising T2* by Imaizumi et al. and Falter et al. demonstrated no statistically significant correlation between gender and the extent of haemosiderin staining. Similar findings were found in this study with females representing 63% of the study population and no statistical correlation with the number of haemosiderin zones (*p* = 0.9).

#### Clinical Severity World Federation Neurosurgical Societies Score

Falter et al. identified a statistically significant relationship between initial WFNS and the extent of haemosiderin staining on T2* imaging (*p* = 0.0008).^12^ This study showed an approaching tendency (*p* = 0.07) between the number of haemosiderin zones and WFNS, but was not statistically significant. The clinical applications imply that haemosiderin detection on SWI in the chronic phase may infer a more clinically severe initial ASAH (clinical WFNS grade).

#### Radiological severity (modified Fisher Score)

Modified Fisher scale on initial NECT has been proven to correlate with vasospasm risk. The mechanism is related to the direct effect of haemorrhage on the vessels in the subarachnoid space.^20^ Imaizumi et al. divided the cerebral convexities into 10 zones and imaged ASAH in the chronic phase. Their findings showed that ≥ 4 haemosiderin zones correlated with an initial modified Fisher score of ≥ 3 (*p* = 0.0005). Falter et al. and Lummel et al. showed that T2* had corresponding statistically significant findings where a modified Fisher score ≥ 3 correlated with the presence of haemosiderin (*p* = 0.0004 and *p* = 0.03, respectively).^12,14^ The mechanism is postulated to be related to the larger initial haemorrhage load resulting in greater haemosiderin staining and hence detection on imaging in the chronic phase. The current study showed no statistically significant correlation (*p* = 0.34). This could be because of SWI’s increased sensitivity to detect small amounts of haemosiderin and thus haemosiderin remotely located from the initial rupture, even in small initial haemorrhage loads (lower modified Fisher scores). As a result of the inherent limitations of this study with a bias of patients with higher modified Fisher scores ≥ 3 (*n* = 35) and underrepresentation of lower modified Fisher scores < 3 (*n* = 5), a true statistical analysis is limited.

#### Location

Aneurysmal subarachnoid haemorrhage in patients with multiple intracranial aneurysms can cause difficulty in identifying the causative ruptured aneurysm and direction of treatment. Ruptured aneurysm localisation rates on NECT and digitally subtracted angiograms vary from 52% to 80%, with studies reporting high sensitivities in localising anterior communicating, anterior cerebral and middle cerebral artery aneurysms.^21,22,23,24^ In these studies, performed in the acute phase, the haemorrhage pattern was identified and causative aneurysm prediction was performed. As the current study was performed in the chronic phase of ASAH, SWI was able to detect haemosiderin distal to the areas of the initial haemorrhage load, thus creating complexity in the localisation of the maximum site of haemosiderin.

**FIGURE 6:**
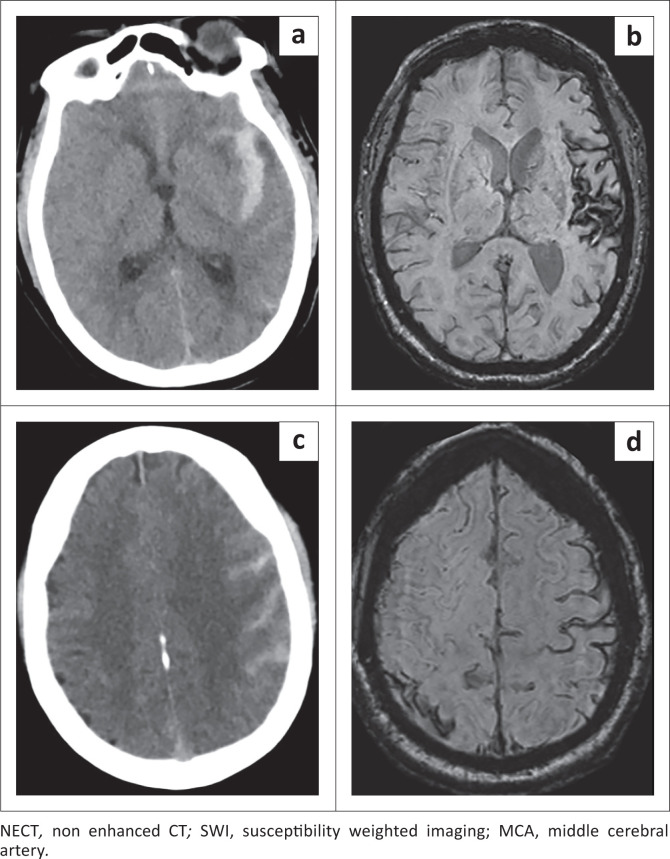
The NECT (a, c) and SWI (b, d) in ruptured left MCA aneurysm. Subarachnoid haemorrhage in the left Sylvian cistern (a) and left frontoparietal convexities (c) and SWI at 3 months demonstrates corresponding blooming in the respective zones (b, d). Of note the maximum haemorrhage load and haemosiderin zone localises to the left Sylvian cistern.

Maximum haemosiderin staining in the Sylvian cisterns was seen with ipsilateral middle cerebral (PPV = 31.25%), posterior communicating (PPV = 50%) and distal internal carotid arteries aneurysms (PPV = 18.75%) and hence was not specific for ruptured MCA aneurysms as observed in previous studies in the acute phase.^21,22,23,24^ This is thought to be related to CSF flow dynamics and deposition of haemosiderin. Figure 6 demonstrates an example of localised haemosiderin and haematoma related to the Sylvian fissure.

Maximum haemosiderin staining in the interhemispheric fissure was limited to anterior communicating (PPV = 85.71%) and pericallosal aneurysms (PPV = 14.29), correlating with previous studies in the acute phase and hence proves useful in the determination of the causative ruptured aneurysm.

Maximum haemosiderin staining in the perimesencephalic cistern was limited to posterior communicating aneurysms (PPV = 100%); however, the known poor sensitivity on SWI in this region because of the base of skull artefact limits its usefulness. Similarly, maximum haemosiderin staining in the posterior cranial fossa cisterns was limited to posterior communicating (PPV = 50%) and posterior inferior cerebellar artery aneurysms (PPV = 50%).

This knowledge may be of use in identifying the site of causative ruptured aneurysm in patients with multiple intracranial aneurysms and help direct appropriate treatment.

### Study Limitations

The small sample size, single centre, retrospective nature of the study and bias with the majority of patients having severe ASAH (modified Fisher ≥ 3) are limitations in this study. Not all initial CT images were available for re-evaluation and information from radiology reports and neurosurgical documentation was relied upon.

Imaging clinically unruptured intracranial aneurysms for occult rupture by assessing for haemosiderin on SWI may be performed in the future to help identify patients requiring treatment.

## Conclusion

Susceptibility weighted imaging is sensitive (95.7%) for the detection of ASAH at 3 months after a single aneurysmal rupture episode and exceeds previous study sensitivities utilising T2* imaging. Sensitivity along the convexity, interhemipsheric fissure, Sylvian cisterns and within the ventricles was better than in the perimesencephic cisterns.

Factors such as age and higher initial clinical severity (WFNS) showed an increased number of haemosiderin zones, while radiologic severity (modified Fisher), site of aneurysmal rupture and gender showed no predictive value in the determination of number of haemosiderin zones. SWI was useful in localisation of the causative ruptured aneurysm, particularly for anterior communicating and pericallosal aneurysms, with haemosiderin in the interhemispheric fissure.

This study proposes that in the clinical scenario of a patient with clinically suspected ASAH without confirmatory imaging in the chronic phase, SWI can be performed to identify previous haemorrhage and possibly identify patients who would benefit from aneurysm treatment.
